# Bio-Inspired Soft Proboscis Actuator Driven by Dielectric Elastomer Fluid Transducers

**DOI:** 10.3390/polym11010142

**Published:** 2019-01-15

**Authors:** Po-Wen Lin, Chien-Hao Liu

**Affiliations:** Department of Mechanical Engineering, National Taiwan University, Taipei 10617, Taiwan; r06522525@ntu.edu.tw

**Keywords:** coiling/uncoiling motions, dielectric fluid transducer, high voltage, compliant electrode, dielectric elastomer, proboscis actuator, soft

## Abstract

In recent years, dielectric elastomer actuators (DEAs) have attracted lots of attention for providing multiple degree-of-freedom motions, such as axial extensions, torsion, bending, and their combinations. The wide applications include soft robots, artificial muscles, and biomimetic animals. In general, DEAs are composed of stretchable elastomers sandwiched by two compliant electrodes and actuated by applying external electric stimuli. Since most DEAs are limited by the breakdown thresholds and low strain-to-volume ratios, dielectric fluid transducers (DFTs) have been developed by substituting dielectric elastomers with dielectric fluids for high breakdown threshold voltages. In addition, DFTs have large rate of lateral extensions, due to their fluid contents, and are beneficial for soft actuators and pumping applications. In this research, we exploited DFTs to develop a soft spiral proboscis actuator inspired by the proboscises of butterflies for achieving uncoiling and coiling motions under external voltages. The bio-inspired spiral proboscis actuator (BSPA) was composed of a coil-shaped tube, a DFT-based pouch, and a spiral spring for mimicking the tubular part, a mechanism to uncoil the tube, and a mechanism to coil the tube, respectively. When applying external voltages to the pouch, the high dielectric fluid was injected into the empty coiled tube for uncoiling where the tube elongated from a compact volume to a stiff and flexible shape. When removing the exciting voltages, the tube retracted to its original coiled shape via the elastic spring. A prototype was designed, fabricated, and examined with high stimulating voltages. It was demonstrated that the proboscis actuator could achieve uncoiling and coiling motions consistently for several cycles. Compared to convection DEA-based pumps with fixed shapes, the proposed actuator is soft and beneficial for portable applications and coiling/uncoiling motions.

## 1. Introduction

With retrospect to evolution through billions of years, nature tends to develop soft and versatile actuators which give creatures more capability to survive and adapt to the dynamic and tough environment. Compared to rotor-based machinery and hard metal components, soft actuators tend to be more tolerant and versatile. In these actuators, numerous motions are achieved according to osmotic pressure, including cnidocytes in cnidarians, capture motions done by carnivorous plants, and guard cells in plants [1].

Soft actuators possess many unique advantages that common mechanical actuators are not able to achieve. These features are extensible, shock-absorbing, lightweight, inexpensive, and highly compliant to the environment. The soft actuator normally performs three kinds of actuating motions, uniaxial stretching [2], bending [3,4], and torsion [5,6,7]. Uniaxial stretching and torsion can achieve great load capacity, but have limited strain. On the other hand, bending motions have great strain performance, but can only bear a limited load. The combination between these types of actuation would create possibilities of multi-degree actuation, however, the complexity of the controllability would increase. Hence, developing a soft actuator using different actuating methods in diversified fields has been an intriguing topic not only for researchers but for business corporations. 

The main application of soft actuators is soft robots. Soft robots are commonly used as industrial grippers [8] to lift fragile and delicate objects. Another common application for soft robots is biomedical rehabilitation assistance. Soft robots that are used in rehabilitation assistance are mainly utilized as artificial muscle [9,10,11], to enhance the life quality for patients suffering from amputation, paralysis, muscle weakness, and degenerative joint disease. Compared to conventional actuators, soft robots can have reduced size and weight, achieving and complying with human-like movements and, at the same, time reduce cost. In past decades, studies of soft robots mainly utilized fluid as the main actuation substance. Both pneumatic and hydraulic actuators have been widely discussed in numerous studies [4,9,12,13,14]. This type of actuation is mainly driven by compressing the fluid to cause pressure difference. These actuators can achieve high actuation force and large strokes, similar to real muscle. However, the main issue of this kind of actuation is that it requires an exterior pump or compressor. The extra machinery would cause more weight and take more space, which may sometimes be too bulky and unrealistic. Another type of soft actuator is shape memory alloys (SMAs). This kind of actuation is induced by a change of temperature and can have a great amount of deformation [15]. However, there are some limitations of SMAs, such as cost, high dependency on temperature, and actuation and de-actuation time [16]. 

The most common type of soft actuator is the dielectric elastomer actuator (DEA) [17,18,19,20,21]. DEAs are materials that consist of one film of dielectric elastomer (usually polymers or silicones) sandwiched by two compliant electrodes. When high voltage is applied, electrostatic stresses (Maxwell stresses) cause the elastomer to compress in thickness and expand in area. In comparison to other actuators (such as motor-based, pressured fluid-based, or SMAs) DEAs have attractive properties, such as large deformation, relatively high speed of reaction, low cost, low elastic stiffness, relatively low weight, and high efficiency [22,23]. In general, a very high electric field is required to generate significant pressures across the elastomer. To develop such high electric fields, the thickness of the elastomer should be small enough, or the applied voltage large enough. Ultra-high electric fields may cause arcing through the air, therefore, the elastomer is generally in the form of a thin film. Although DEAs have a high maximum strain and high efficiency, material properties make it vulnerable to dielectric breakdown. Once the dielectric breakdown happens, the breakdown material would suffer from irreversible property change. The damaged polymer would form an ionized easy path for electric current, thus, would significantly drop the breakdown voltage. Moreover, the chance of breakdown would increase when the electrode is scaled up, which reduces the reliability and potential for greater scale applications [24].

Hence, later researchers developed a self-healing actuator [25] using the same actuating principle of using electrostatic force, but replacing the insulator from dielectric film to dielectric liquid. Researchers called this a dielectric fluid transducer (DFT). This kind of liquid-based actuator improves the permanent breakdown disadvantage of DEAs, and has the same actuation properties as the DEAs. Previous research shows that regarding the applications of DFTs, they could be used as a pump [26], optical lens [27,28], and linear actuator [25,29]. These applications showed the potential of the DFT, but there are limitations of these mentioned actuation motions, which have their drawbacks. The elongation of the actuation is usually perpendicular to the activation direction of the electrode. Thus, the strain of the actuation would be relatively small. Research performed by Kellariss et al. [29] showed that the usage of an actuator array would increase strain. However, the strain compared to the overall size of the actuator was still only 5%. Furthermore, the retraction of the actuation is highly reliant on the exerted weight. 

Here, we introduced the bio-inspired spiral proboscis actuator driven by a dielectric fluid transducer. The actuator movement was inspired by butterfly proboscis movements, and our goal is to achieve proboscis-like coiling and uncoiling movement [30]. The BSPA actuator has the following features: (i) the actuator can actuate without any rigid component or metallic parts; (ii) it can perform under large strain with a relatively small overall size; (iii) it can be made from inexpensive materials with an industrialization-ready fabrication method and can be easily mass produced; (iv) it can be made from a variety of materials that differ for applications and are not limited to materials mentioned in this article; and (v) no external load or pre-activate motion is needed. The spiral proboscis actuator is composed of two sections, the pouch section acting as the liquid pump and the spiral proboscis tube serving as the actuator. When the voltage was applied, the pouch would contract, causing the pressure to build. The inner pressure would force the liquid to flow into the tube, making the tube extend. When the voltage was removed, the pouch would expand to its original size, and the liquid in the tube would flow back to the pouch. The attached spiral spring then retracted the tubing to its original spiral shape. The actuator we proposed has the pouch length-to-strain ratio of 1:0.76, and it can perform a complete stroke without any exterior weight or pressure assistance. 

This paper is organized as follows. In the next section, we present the working principle of BSPA based on DFT and applied external voltages. Then, the design and dimensions are described. Subsequently, the fabrication processes and the selections of materials are disclosed. Then, high voltage experiments are conducted to examine the coiling and uncoiling motions of the fabricated BSPA. Finally, some special properties and phenomena observed in the experiments are discussed. Important results are summarized at the end of the paper.

## 2. Working Principle 

**Figure 1:**
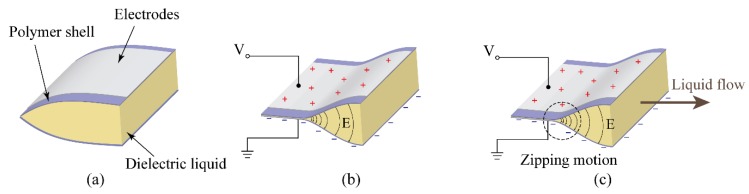
Illustrations of the zipping motion. (**a**) Without external voltage, the dielectric fluid transducer (DFT) is in de-activate mode, where the pouch is full of dielectric liquid. (**b**) When applying external voltages, the electrostatic forces caused by the compliant electrodes squeeze the pouch to force the dielectric liquid flow to the empty tube. (**c**) As the thickness decreases, the zipping motion occurs at the electric field concentrated area.

In this research, we exploit dielectric fluid transducers (DFTs) to achieve spiral proboscis-like actuations. The BSPA is driven by electrostatic forces due to the applied external voltages to pump the dielectric liquid into the empty tube, to uncoil the tube and transduce its energy. The BSPA converts the electrical energy into mechanical energy to achieve the desired movements. The BSPA consisted of dielectric fluid encased by flexible polymer shells (low-density polyethylene (LDPE)), forming a liquid pouch, as shown in Figure 1a. A tube made with the same material of the pouch is extended out, serving as a transducer. While liquid pressure is formed within the pouch, the dielectric liquid is injected into the tube, making it expand and extend. A spiral spring made of a non-metal elastic material (polyethylene terephthalate (PET)) is attached to the tube, giving the tube the capability to retract after being extended.

Electrodes are attached on the top and bottom layers of the pouches. When the high voltage is applied, the top and bottom electrodes will accumulate positive and negative charges, forming strong electric fields between two electrodes. When the electric potential increases, the electric field also increases. Furthermore, the electric fields on the edge will concentrate, due to the geometric shape of the pouch shown in Figure 1b. Figure 1c shows that the concentration of the electric fields can cause the upper and lower layer to attract and adhere to each other, pushing dielectric liquid to the other sides of the pouch. The adhering motion and shrinkage section move along the concentrated electric fields. This motion is called zipping motion. 

Due to the compliant electrodes, the Maxwell stress due to the electrostatic force is expressed as [31]

(1) $$\sigma =\frac{1}{2}\varepsilon_{0}E^{2}.$$

Based on the Maxwell stress (σ = *P*/*A*), the accumulated pressure *P* is derived as

(2) $$P=\frac{1}{2}\varepsilon_{0}AE^{2},$$

where *P* represents the pressure build-up inside the DFT, while the $\varepsilon_{0}$ represent dielectric constant of the fluid between two electrodes. *A* represents the area of the electrodes and *E* represents the magnitude of the electrical field inside the pouch. To increase the accumulated pressure, we need to find dielectric liquid with greater $\varepsilon_{0}$ and increase the electrical field, while the electrical field has a greater impact on the pressure. 

**Figure 2:**
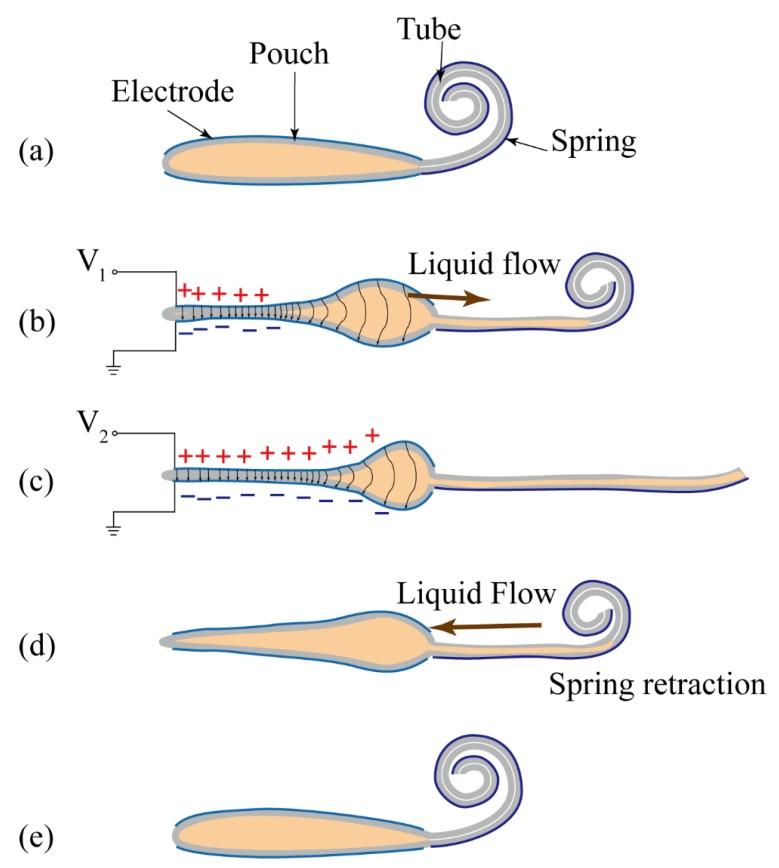
The coiling and uncoiling motions of the proboscis actuator. (**a**) The original shape of the actuator under no external voltages. (**b**) When applying external voltage, *V_1_*, the DFT forces liquid flow into the empty tube to uncoil the tube. (**c**) As the voltage increases, the soft tube flattens with a flexible and stiff shape. (**d**) When removing the external voltages, the tube coils due to the elastic spring. (**e**) The actuator returns to its original shape.

Figure 2a–c present actuation motions (i.e., uncoiling motions) of the actuator. The pressure built up in the pouch caused by the electrostatic force squeezes the dielectric liquid out of the pouch and into the tube. The originally empty tube is filled with dielectric liquid. The pressure inside the tube will cause the tube to expand and solidify compared to its original softness, making the cross-section transfer from flat to oval-like shape. The solidification process will cause the original soft and spiraled tube to be extended and straightened. Figure 2d–e show the retraction motion caused by removing the external voltages. The tube coils due to the elastic spring. 

In the research conducted by Giacomo et al. [32], the global energy balance on the system can be written as follows:

(3) $$dE_{m}+dE_{e}=dU_{m}+dU_{e},\;$$

where $dE_{m}$ is the work done by the external load which, in this case, is zero. $dE_{e}$ is the energy supplied by the electronics, $dU_{m}$ is the variation in elastic potential energy stored in the device, and $dU_{m}$ is the electrostatic potential energy stored in the device. The pouch is made out of flexible but inextensible film, in which we can observe that, in the absence of any voltage, the uniform pressure inside the pouch would push outward, making the thin film wall a portion of a cylinder. Thus, we can assume that the outline of the inactive pouch is a portion of a circle.

The second assumption is that, before reaching enough pressure, the liquid inside the pouch will not inject into the tube but stay inside the pouch, as shown in following figure. Thus, we can assume that the liquid inside the pouch remains at a constant volume, $\Omega_{f}$. $\Omega_{f}$ can be found by geometry relation, as follows.

(4) $$\Omega_{f}=2\left(r^{2}\tan^{-1}\left(\frac{a}{2r-2h}\right)-\frac{a(r-h)}{2}\right)$$

**Figure 3:**
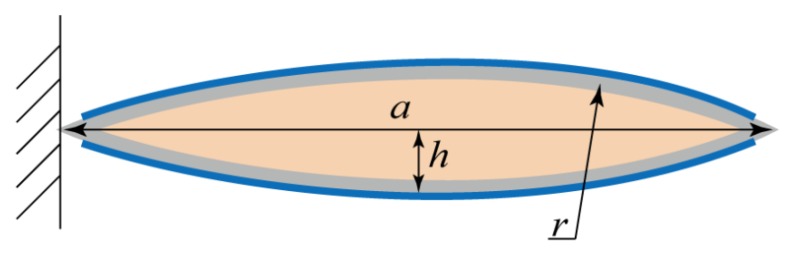
The cross-section view of the pouch with the original shape shown in Figure 2a. We assumed that the liquid inside the pouch remains a constant volume, and the outline of the pouch maintains a portion of a circle, where *a* is the lateral distance and *h* represents the half-thickness of the pouch. *r* is the radius to the outline of inactive pouch, and the electrode is attached on the whole length of the pouch.

**Figure 4:**
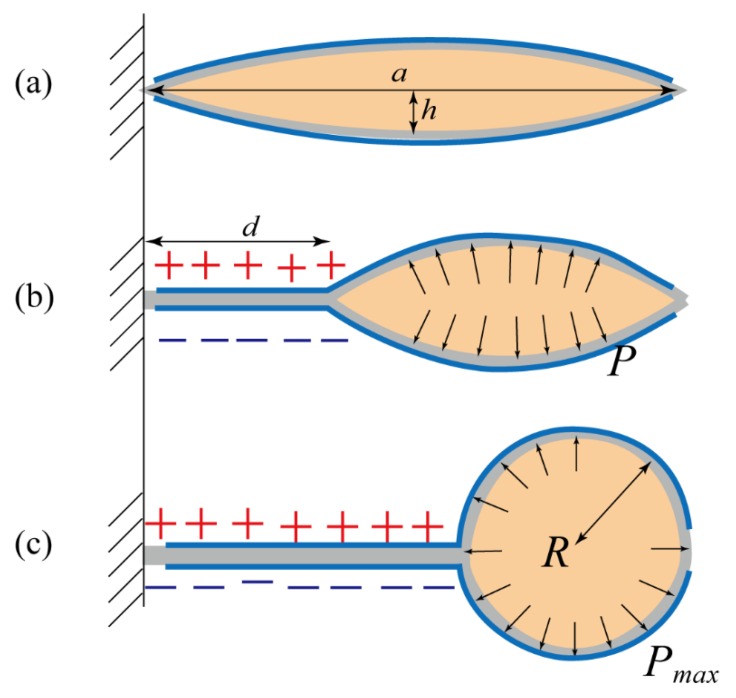
Illustrations of squeezing the pouch and pumping the dielectric liquid into the tube. (**a**) The original shape without external voltages. (**b**) When applying external voltages, the electrostatic forces between two compliant electrodes squeezed the pouch with an active length of *d*. Then, the dielectric liquid was pushed to the inactive region, and accumulated with the liquid pressure, *P*. (**c**) If the applied voltage was above a certain threshold, all dielectric liquid was pushed to the end of the pouch with a circular area, *R*, and the generated liquid pressure, $P_{max}$, was sufficient to uncoil the tube.

The dimensions *a*, *r*, *h* can be found in Figure 3. While the voltage is given, the electrodes adhere and zip together, changing the geometry of the pouch as shown in Figure 4. The outline of the pouch still maintains a portion of the circle with the radius *r*. As shown in Figure 4c, due to the geometry, the fully compressed radius, *R*, can be written as the following.

(5) $$R=\sqrt{\frac{\Omega_{f}}{\pi }}$$

We neglected the collapsed volume under the adhered electrode. We assumed that in order to form a circular pressure-concentrated area R, shown in Figure 4c, the applied voltage needed to be large enough. The threshold voltage will be discussed later, where the threshold voltage is 10 kV. We assumed that only by forming the entire circle, *R*, can we induce enough pressure, $P_{max}$. The $P_{max}$ is defined as the pressure sufficient to inject liquid into the tube shown in Figure 4c. The pressure $P_{max}$ could be calculated via Equation (7), assuming that the transducer capacitance is distributed only by the electrode adhering region. Henc, the transducer capacitance C can be written as follows.

(6) $$C=\frac{\varepsilon_{p}wd}{2t_{p}},\;$$

where *w* is the transducer width, *d* is the adhered electrode length, and $t_{p}$ represents the thickness of the polymeric film.

Then, we can derive the pressure inside the pouch as follows.

(7) $$P=\frac{dU_{el}}{d\Omega_{f}}-\frac{V^{2}}{2}\frac{dC}{d\Omega_{f}},\;$$

where $U_{el}$ represents the elastic energy, which can be calculated by the strain energy density function mentioned in the research done by Giacomo et al. [32]. The retracting motion depended on the PET spiral spring. After the voltage was removed, the zipping effect would disappear, making the pressure inside the pouch decrease. The tube could then be retracted by the PET spring. 

## 3. Design

**Figure 5:**
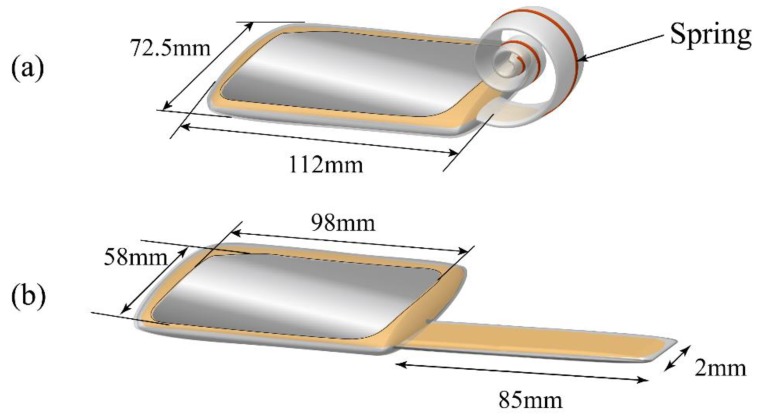
The physical dimensions of the proboscis actuator for (**a**) coiling and (**b**) uncoiling states, where the dimensions of the pouch and tube are 112 mm × 72.5 mm and 85 mm × 2 mm, respectively. The area size of the electrodes is 98 mm × 58 mm.

The BSPA was composed of a pouch made of polyethylene, an empty tube, and elastic springs, as shown in Figure 5. The pouch was designed with the dimensions of 112 mm × 72.5 mm for providing sufficient liquid pressure, due to the contained dielectric liquid, to uncoil the tube. The compliant electrodes had an area size of 98 mm × 58 mm for providing electrostatic forces. The tube was designed with the dimensions of 85 mm × 2 mm for pumping dielectric liquid to flatten the tube. The detailed dimensions of the device are shown in Figure 5.

## 4. Fabrications

**Figure 6:**
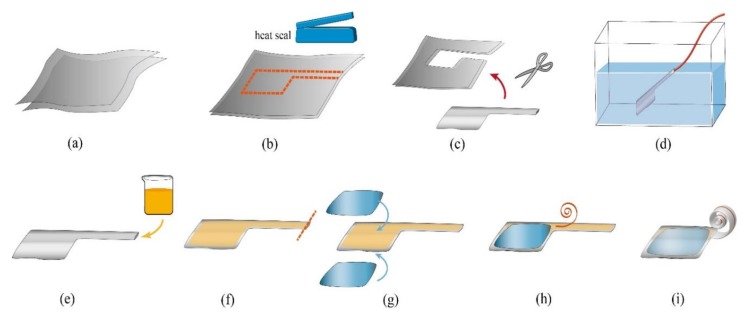
The fabrication of the bio-inspired spiral proboscis actuator (BSPA) actuator. (**a**) Two thin low-density polyethylene (LDPE) films were prepared. (**b**) The pattern was drawn on the LDPE film, and marked with Kapton tape. The Kapton tape serves as a marker and protection for the areas we do not want the heat to affect. The heat seal process was done using a commercial heat seal machine. (**c**) After heat sealing, the extra LDPE film was then cut away from the actuator. (**d**) The device was submerged under water to test the air-tightness of the seal. (**e**–**f**) The actuator was taken out of the water, allowed to dry, and filled with dielectric liquid. (**g**) After heat sealing the opening of the tube, the electrodes were attached on the top and bottom of the pouches. (**h**) The PET spring was attached to the tube by tape. (**i**) The final device.

In this research, we fabricated the actuators according to the fabrication process shown in Figure 6. The actuator was composed of three main segments using different materials. The outer shell was made of 0.2 mm thick low-density polyethylene (LDPE), commercially available and the most commonly used polymer material, in our case, on a daily basis. The polyethylene had low hardness, low rigidity, low friction between layers, and had high ductility, making it a good material for making film and food packages. Meanwhile, LDPE had a good electrical breakdown strength of 79 kV/mm [33]. The availability, softness, and good electrical breakdown strength was the reason why we chose low-density polyethylene (LDPE) as our outer shell. 

The dielectric liquid inside the shell was isopropyl myristate (IPM), a skin-safe oil commonly used in cosmetic creams and body lotions. IPM had high breakdown strength and a relatively high dielectric constant of 3.124 (at 313.2 K) [34] and low viscosity of 6.5 cP [35]. In order to pump the dielectric liquid and let the liquid flow freely in the narrow tube, the lower viscosity of the dielectric liquid was important. The low viscosity property made IPM oil suitable for use in injection motion in this research.

The fabrication process started with the heat seal process. Two layers of PE sheet were cleaned with compressed air to get rid of any kinds of dust or debris which would cause damage to the structural integrity and lead to leakage of the pouch. The two LDPE films were placed one on top of the other. We carefully aligned the two LDPE films and fixed them together by applying Kapton tape. The Kapton tape also acted as a marker and protection for the areas we did not want the heat to affect. Then, we heat-sealed the two LDPE films using a 300 W impulse heat sealer, one stroke at a time, to form the pouch and the tube. The pouch was used as a reservoir for the dielectric liquid, and the tube acted as the actuator. We filled the finished product with compressed air, then pressed it under water to test whether there was any leakage or not. This process ensured the air-tightness of the entire structure. The device then was taken out of the water and dried, and all the edges were trimmed off carefully. Next, we injected the IPM dielectric liquid through the top opening using a thin liquid dropper. The amount of IPM dielectric liquid injected was 25 mL. After injection, the top opening would be heat-sealed using the same process mentioned in the previous article. After filling the dielectric liquid, the electrodes were attached to the upper and bottom layers of the pouch. The electrodes were made of aluminum foil with a thickness of 16 μm, and were 98 mm × 58 mm in size. The fillet radius of the electrode was 5 mm on each corner, to prevent concentration of the electric field on the sharp edges. 

The reason why the electrodes were offset 7 mm to each side of the pouch was to prevent any potential electrical arcing passing through air or through LDPE shell. The potential of arcing through the air gap and through PE shell will be discussed later. Under normal conditions at atmospheric pressure, air served as an excellent non-conductor, but when the applied voltage was high enough, the air would be ionized and transform from non-conductor to conductor. The electrical breakdown also happened in the dielectric material. In dielectric material, such as LDPE film, the high voltage would create a permanent low resistance path within the material. The sudden strong current would cause irreversible failure to the material. 

**Figure 7:**
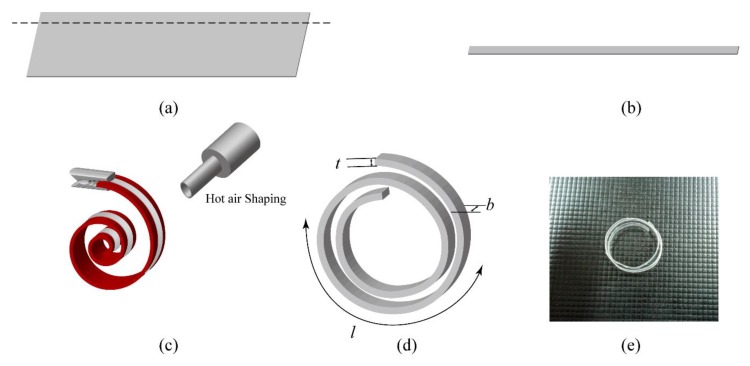
The fabrication of the spiral spring. (**a**) A thin film of PET with the thickness of 25 μm was prepared. (**b**) A thin strip was cut from the film and clamped on an acrylonitrile butadiene styrene (ABS) mold. (**c**) The hot air shaping process. The PET would reach permanent deformation after exceeding 70 °C. (**d**) The spiral spring was cooled off on the mold to retain its deformation. (**e**) A photograph of the fabricated spring.

After attaching the electrodes, the spiral spring would be attached to the tube. The spiral coil spring had a linear load response and was suitable for low loads [36]. The spiral spring was made out of (polyethylene terephthalate (PET) film with a thickness of 25 μm. PET is a common material used in clothing fibers, containers for liquids and foods. The main advantage of PET is its thermosoftening property. The PET has great plasticity when heated and solidifies upon cooling which makes it a great material for spring shaping. As shown in Figure 7, the PET film was cut into strips, with a length of 100 mm and width of 1 mm. The strip then was pre-formed into a spiral shape by fitting into the 3-D printed acrylonitrile butadiene styrene (ABS) mold using a heat gun to achieve heat shaping, and cooled down to retain its spiral shape. The rigidity formula of the spiral spring [37] is mentioned in the following equation, and the detailed values are shown in Table 1.

(8) $$k=\frac{\pi Ebt^{3}}{2160L}\;(N\;\times \;\mathrm{mm}/\mathrm{degree})$$

**Figure 8:**
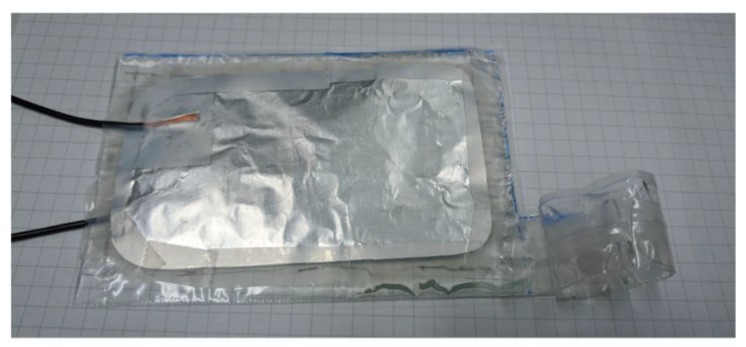
Photograph of the fabricated BSPA actuator.

The final device is shown in Figure 8, and the designed zipping area is equal to the area of the electrodes. However, due to the pressure accumulation shown in Figure 4c, the adhered area would not be the whole electrode. 

## 5. Experiments and Results 

**Figure 9:**
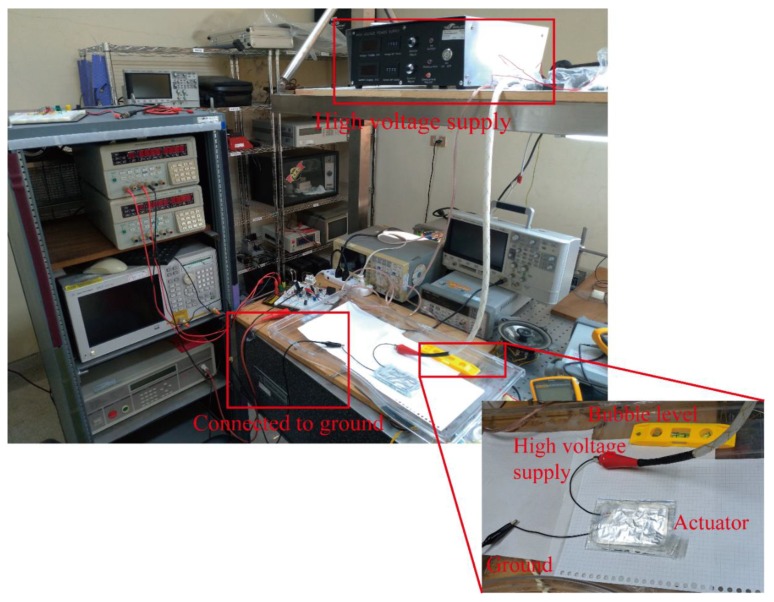
The experimental setup for the BSPA.

**Figure 10:**
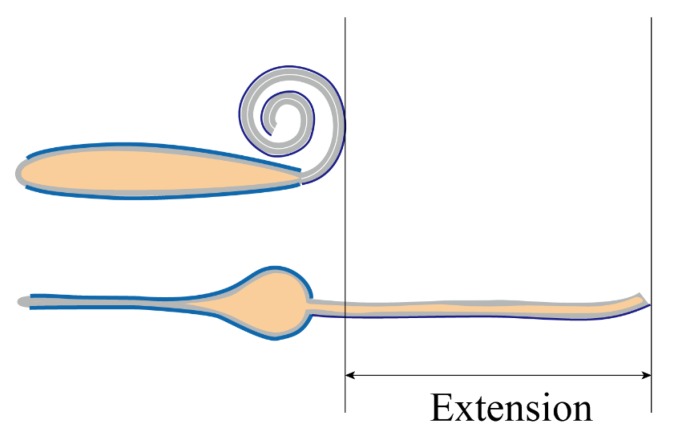
Illustration of the extension of the BSPA.

Figure 9 shows the experimental setup for examining the fabricated BSPA with high voltages. The fabricated BSPA was placed flat on an insulated plastic tray for shielding high voltages. The tray was set parallel to the horizontal surface of the table, to avoid any tilting or gravity effect for dielectric liquid, using a bubble level. Two individual copper wires were attached to the compliant electrodes on the top and bottom sides of the BSPA. The adjustable high voltages were supplied by a high voltage supply (model: FALCO). We applied a square voltage pulse wave with different amplitudes to the BSPA to examine its uncoiling and coiling motions. To measure the time-varying uncoiling and coiling motions, the camera was exploited to take videos of these motions with 30 fps (frame per second). In each time frame, the extension of the actuator could be measured. Figure 10 illustrates the measurement of extensions of BSPA, from the end edge of the coiled tube with the original shape to the end edge of the tube during uncoiling processes, when applying external voltages.

**Figure 11:**
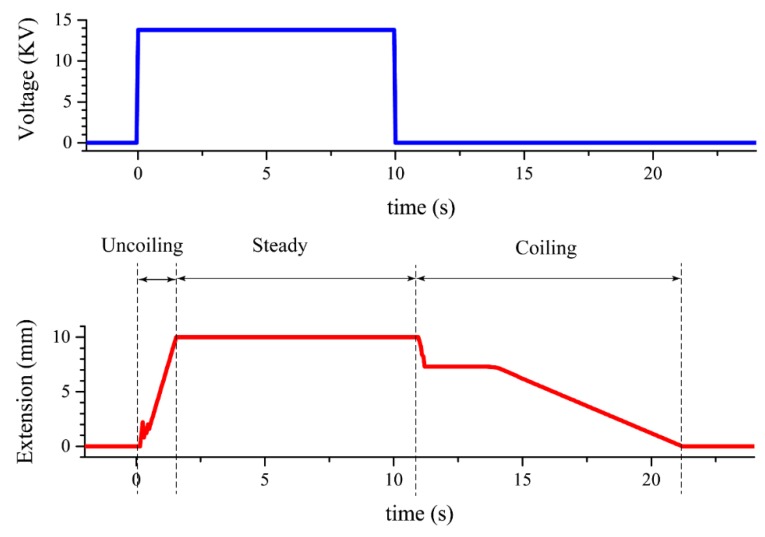
The actuation response and the corresponding applied voltage in time domain. When applying external voltages, the actuator uncoiled linearly with time and extended to its maximum length. When removing the voltage, the actuator coiled to its original shape via the elastic spring.

According to Equation (2), the dielectric pressures were proportional to the square of the electric fields ($P\propto E^{2})$ [31], where the magnitudes of electric fields depended on the applied voltages. In other words, manipulating the applied voltages could provide different liquid pressures to uncoil the tube to obtain different extensions, shown in Figure 11. 

Based on empirical experiments and trial-and-error tests, we discovered that the breakdown voltage of our setup was approximately 14.5 kV. Then, we scaled down the applied voltage to 13.8 kV as our operation voltage, to avoid occurrences of breakdown. The thickness of the pouch before applying any voltage was 4.1 mm. When applying 13.8 kV between two compliant electrodes, the electric field, E, could be calculated by a simple equation U=E/d (V/m), where U represented the electrical potential and d was the distance between two electrodes. The estimated trigging electric field was 3.365 MV/m. 

Figure 11 shows the extension response and the corresponding applied voltage in time domain. When the voltage was applied, the extension showed a temporary fluctuation during 0 to 0.6 s, and the fluctuation was due to the fact that the pouch was in its zipping motion, which made the pressure slightly unstable. After the fluctuation period, the pressure built up in the pouch, and pumped the dielectric liquid into the tube. The actuator uncoiled linearly with time and extended to its maximum length. The actuation had a fast response of 10 cm per 1.5 s.

After removing the applied voltage, the electrons were still stagnant on the electrodes, causing the electrodes to still stick to each other. Without any external electrostatic force, we had to wait until the electrons diffused away so, in reality, the retraction rate (i.e., coiling motion) would be slower compared to the extension rate, as shown in Figure 11. In this experiment, the retraction time was about ten times slower than the extension time. In the retraction step, the spiral coil spring acted as the retractor. To shorten the retraction time, the greater spring constant could be used, however, the strengthening of the spring would make the extension harder. The stiffer the spring was, the higher the voltage that must be applied in order to create enough pressure, which could lead to a potential electrical breakdown. 

In order to pump the dielectric liquid to the empty tube and let the liquid flow freely in the narrow tube, the thickness of the liquid was the main concern. The dielectric constant mentioned in the previous section also gained a foothold. Since the dielectric constant and the viscosity of the liquid mattered, we tested some other commercially available dielectric liquids and insulating oils, as shown in Table 2. We tested the feasibility of different viscosities and dielectric constants by changing the dielectric liquid inside the pouch, and we then chose IPM as our dielectric liquid, due to its extremely low viscosity and relatively high dielectric constant. 

**Figure 12:**
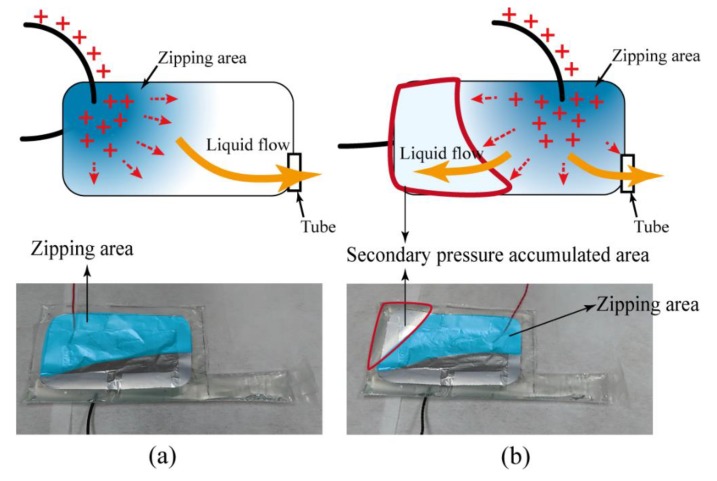
The effect of the locations of the copper wires. (**a**) If the copper wires were placed away from the tube, the actuation started at the bottom and pumped the liquid into the tube. (**b**) If the copper wires were placed near the tube, only a portion of liquid was pumped into the tube, and the rest was squeezed away from the tube. This formed a secondary pressure-accumulated area and significantly degraded the uncoil motion of the tube.

The placements of copper wires could affect the locations where the zipping motion started. Figure 12 shows two different attached locations of the wires. While flowing through the wire, the electrons would accumulate in the near field first, and then spread to the whole electrode. The accumulated electrons caused electric field concentrations, where the zipping motion started. The different placements of the wires would cause different starting points. As shown in Figure 12, the undesired placements of the wires would form a secondary pressure-accumulated area inside the pouch. This indicated that only a portion of liquid was pumped into the tube, and the rest was squeezed into the secondary pressure-accumulated area. This reduced the pumping liquid needed for uncoiling actuation, and made the usage of electric potential energy inefficient. Thus, the desired starting point and location to place the wires was at the end of the pouch. 

**Figure 13:**
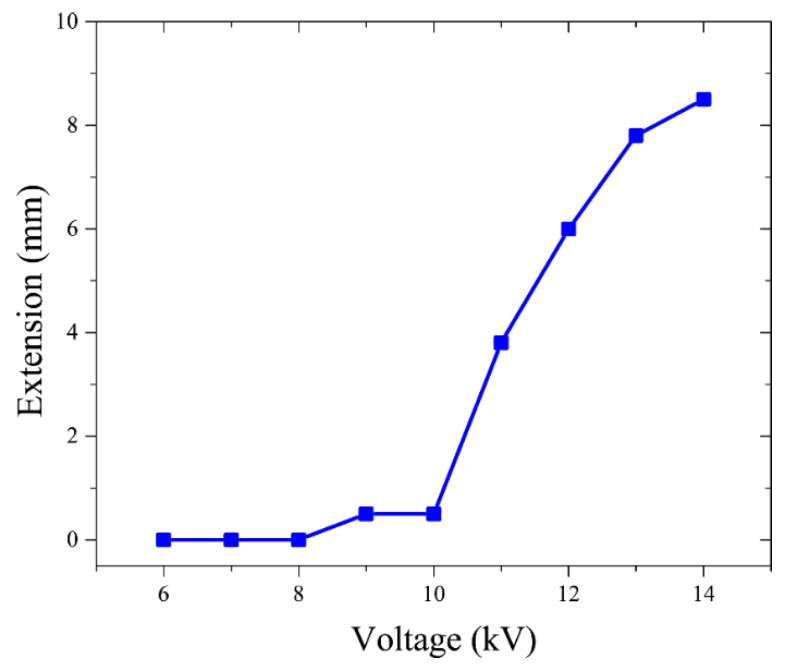
The extension response of the BSPA for different external voltages. The lowest threshold voltage of uncoiling motion was 10 kV, and the actuator flattened completely at 13.8 kV.

The actuations of the BSPA depended on the magnitude of the applied voltages. To find the threshold voltage for trigging the uncoiling motions, several experiments were conducted with the same pouch and experimental setup, via adjusting the applied voltages from low to high values, shown in Figure 13. The applied voltage varied from 6 to 14 kV, with a 1 kV interval. If the voltage was in the range of 6–10 kV, the extensions were almost marginal, indicating that the electrostatic forces due to the applied voltage were insufficient to generate enough liquid pressure to uncoil the empty tube. The lowest threshold voltage of uncoiling motion was 10 kV. When the applied voltage was above 11 kV, the accumulated liquid pressures were enough to extend the tube, and caused actuations. As the applied voltage increased, the extension of the actuator increased. However, the extension was not proportional to the square of the applied voltage, since we did not take into account the effects of the weights of the dielectric liquid, the resistances of the empty tubes, and the viscosity of the dielectric liquid. The BSPA flattened completely at 13.8 kV, where the tube had a stiff and flexible shape. As mentioned before, breakdown occurred when the voltage was above 14 kV. 

**Figure 14:**
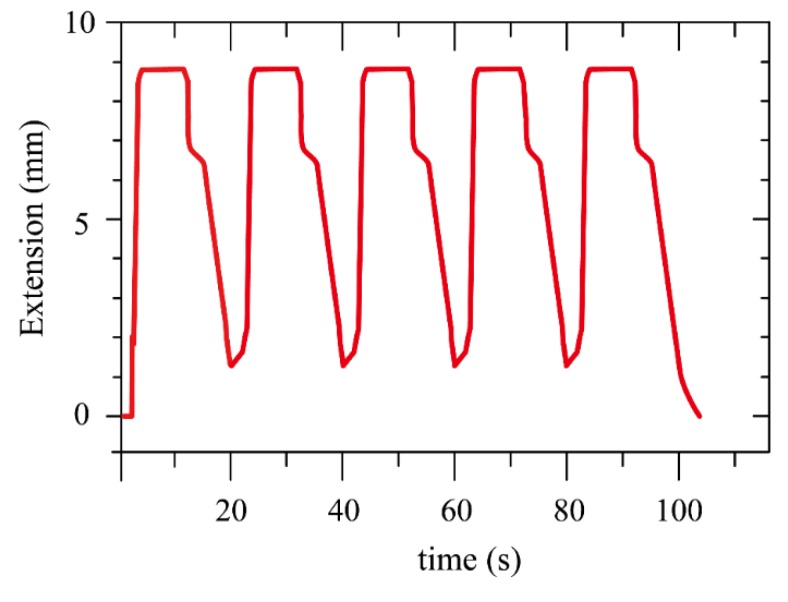
The extension response of the BSPA during several cycles. Within each period, the actuator achieved uncoiling and coiling motions consistently. Note that the actuator did not return to its original shape because the coiling motions needed more time to complete the retraction processes.

To examine the consistency and repeatability, the BSPA was tested with several cycles via a pulse train signal, and the extension response was shown in Figure 14. Within each period, the BSPA uncoiled and coiled consistently. Note that the actuator did not return its original shape (i.e., the extension was not 0) because the coiling motions needed more time to complete the retraction processes.

## 6. Discussions

**Figure 15:**
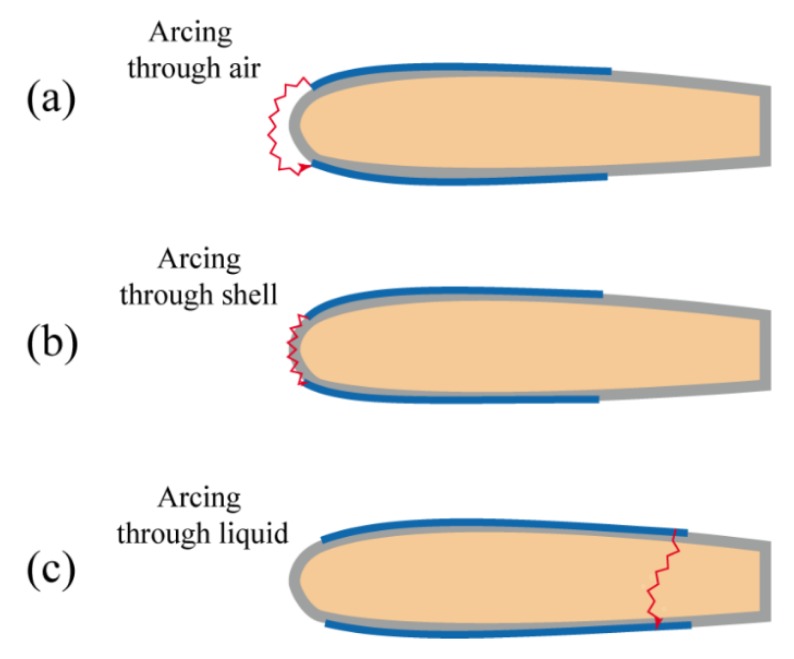
The potential arching paths through the air shell and dielectric liquid.

Dielectric breakdown, in general, could damage the dielectric structures or fail the actuations of our device. Dielectric breakdown was a statistical process where breakdown happens at any voltage level around the threshold voltages [46]. To avoid breakdown, the operation voltage should be lower than the breakdown threshold voltage. Figure 15 illustrates three potential arching paths that happened in our device, including arcing though air, arcing through shell, and arcing through liquid. Since the breakdown threshold voltage of dielectric liquid was lower than that of the polymer, arcing through dielectric liquid tended to occur more often. In our experiments, when the applied voltage was above a certain level, three arcing situations could be observed. 

Compared to non-liquid elastomers, one advantage of utilizing dielectric liquid was the self-healing effect that occurred during breakdown [25]. The self-healing effect was that, after breakdown occurred, the ionized areas of dielectric liquid due to breakdown would be fulfilled with nearby dielectric liquid, where the entire dielectric liquid remained insulated. Therefore, the dielectric liquid could be reused, even though breakdown occurs within the liquid.

For most DEAs, carbon powders had been widely used as stretchable compliant electrodes for large deformations [47,48,49,50]. Since the compliant electrodes of the pouch of the BSPA remained the same, we exploited commercially available aluminum foil as a compliant electrode in this research. One advantage was that the pre-process of flat compliant electrode was relatively simple, by just flattening any bumps or dents to prevent concentration of the electric field. For comparison, we fabricated conducting compliant electrodes composed of carbon powder paste. We mixed 1000 mesh graphite powder with deionized water and water-based PVA glue to form the carbon powder paste. 

**Figure 16:**
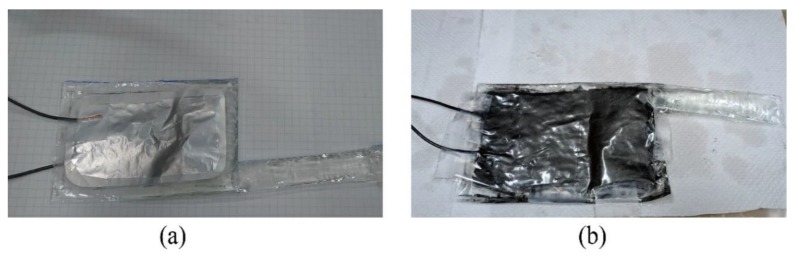
Comparisons of compliant electrodes made of (**a**) aluminum foil and (**b**) carbon powder paste.

Figure 16 shows the comparisons of two different compliant electrodes composed of aluminum foil and carbon powder paste. Although both could squeeze the pouch and uncoil the tube, the carbon powder-based electrodes tended to break down due to rough surfaces. On the other hand, aluminum foil-based compliant electrodes could achieve a relatively smooth surface. 

## 7. Conclusions

In this research, we presented a soft spiral proboscis actuator, inspired by the proboscises of butterflies, for providing uncoiling and coiling motions under external voltages. The spiral proboscis actuator was composed of a coil-shaped tube, a pouch, and a spiral spring for mimicking the tubular part, a mechanism to flatten the tube, and a mechanism to retract the tube, respectively. When applying external voltages to the dielectric fluid transducer-based pouch, the high dielectric fluid was injected into the coiled tube to uncoil where the tube elongated from the small volume to the stiff and flexible shape. When removing the exciting voltages, the tube retracted to its original coiled shape via the elastic spring. A prototype was designed, fabricated, and examined with high stimulating voltages. It was demonstrated that the proboscis actuator could achieve uncoiling and coiling motions consistently for several periods. The research results are expected to be beneficial for soft actuators and coiling/uncoiling motions.

## Figures and Tables

**Table 1:** The geometric parameters of the spiral spring.

Parameters	Meaning	Value
*E*	Spring elasticity	2.76 GPa [38]
*b*	Spring width	1 mm
*t*	Spring thickness	0.25 mm
*l*	Spring length	100 mm

**Table 2:** Different types of dielectric liquid and their properties.

Liquid Type	$\mathrm{Dielectric}\;\mathrm{Constant}\;\varepsilon_{0}$	Viscosity
Castor Oil	1.57–4.01 [39]	2400 cP [40]
Silicone Oil	2.73 [41]	100 cSt (by datasheet)
Linseed Oil	3.17–3.35 [42]	33.1 cP [43]
Olive Oil	3.06–3.08 [44]	63.5 cP [45]
Isopropyl Myristate (IPM)	3.124	6.5 cP
